# RFX4 is an intrinsic factor for neuronal differentiation through induction of proneural genes POU3F2 and NEUROD1

**DOI:** 10.1007/s00018-024-05129-y

**Published:** 2024-02-22

**Authors:** Wonyoung Choi, Mu Seog Choe, Su Min Kim, So Jin Kim, Jiyeon Lee, Yeongun Lee, Sun-Min Lee, So Hee Dho, Min-Young Lee, Lark Kyun Kim

**Affiliations:** 1 Interdisciplinary Program of Integrated OMICS for Biomedical Science, The Graduate School, Yonsei University, Seoul, Korea; 2 College of Pharmacy, Research Institute of Pharmaceutical Sciences, Kyungpook National University, Daegu, Republic of Korea; 3 Department of Biomedical Sciences, Graduate School of Medical Science, Brain Korea 21 Project, Gangnam Severance Hospital, Yonsei University College of Medicine, Seoul, 06230 Republic of Korea; 4 Department of Physics, Konkuk University, Seoul, Republic of Korea

**Keywords:** RFX, Proneural factor, Epigenome, Neuropsychiatric disorders, Neuronal differentiation, Stem cell

## Abstract

Proneural genes play a crucial role in neuronal differentiation. However, our understanding of the regulatory mechanisms governing proneural genes during neuronal differentiation remains limited. RFX4, identified as a candidate regulator of proneural genes, has been reported to be associated with the development of neuropsychiatric disorders. To uncover the regulatory relationship, we utilized a combination of multi-omics data, including ATAC-seq, ChIP-seq, Hi-C, and RNA-seq, to identify RFX4 as an upstream regulator of proneural genes. We further validated the role of RFX4 using an in vitro model of neuronal differentiation with RFX4 knock-in and a CRISPR-Cas9 knock-out system. As a result, we found that RFX4 directly interacts with the promoters of POU3F2 and NEUROD1. Transcriptomic analysis revealed a set of genes associated with neuronal development, which are highly implicated in the development of neuropsychiatric disorders, including schizophrenia. Notably, ectopic expression of RFX4 can drive human embryonic stem cells toward a neuronal fate. Our results strongly indicate that RFX4 serves as a direct upstream regulator of proneural genes, a role that is essential for normal neuronal development. Impairments in RFX4 function could potentially be related to the development of various neuropsychiatric disorders. However, understanding the precise mechanisms by which the RFX4 gene influences the onset of neuropsychiatric disorders requires further investigation through human genetic studies.

**Supplementary Information:**

The online version contains supplementary material available at 10.1007/s00018-024-05129-y.

## Introduction

The precise regulation of crucial genes that determine cell fate during cellular differentiation is essential [1, 2]. One way to achieve this is by controlling epigenetic changes which directly modulate chromatin states to control gene expression [3–5]. Transcription factors (TFs) that bind to chromatin regulatory regions also play a crucial role in regulating gene expression [6]. Lineage-specific TFs regulate a set of development-related genes [7]. For proper neuronal development, there are important roles of neuronal lineage-directing factors, known as proneural factors, which were first discovered in Drosophila [8]. Since the discovery of proneural factors, several studies have attempted to find an optimal combination of TFs for the efficient production of functional neuronal cells [9–11]. BAM factors, which comprise POU3F2, ASCL1, and MYT1L, play important roles in determining neuronal lineages from pluripotent stem cells and in direct reprogramming from dermal fibroblasts to functional neurons [12, 13]. In addition, NEUROD1 can induce neuronal differentiation and has synergistic roles in neural fate-determining processes with the co-expression of BAM factors [13, 14]. However, although there have been numerous studies on the external factors that induce neuronal differentiation, little is known about the factors that intrinsically induce the expression of these proneural factors. The research on the neural differentiation mechanism has been enhancing our understanding of neuropsychiatric disorders originating from various neurodevelopmental origins, which include schizophrenia, depression, autism spectrum disorder, and bipolar disorders [15]. Especially, various in vitro models are developed with iPSC to investigate the molecular pathogenesis of neuropsychiatric disorders [16]. Through epigenomic analysis in such models, we can define transcription factors, or their target genes, associated with the onset of neuropsychiatric disorders.

In this regard, we used multi-omic epigenome data from iPSC and iPSC-derived neural progenitor cells (NPCs) to identify the up-stream regulator of proneural genes. As a result, RFX4 were found to be directly bound to promoters of *POU3F2* and *NEUROD1,* also bound to NPC-specific enhancer which target those proneural factors. Those enhancers which bound by RFX4 were found to be highly associated with the neuropsychiatric disorders by disease ontology analysis. The function of RFX4 in neuronal development were validated with in vitro model, which includes overexpression and CRISPR-Cas9 KD of RFX4 gene. Finally, transcriptomic analysis of RFX4 KD in vitro model, we could confirm the possible role of RFX4 on the development of various neuropsychiatric disorders.

## Materials and methods

### Human pluripotent stem cell culture

Human-induced pluripotent stem cells (hiPSCs) derived from fibroblasts were obtained and approved by the Institutional Review Board of Yonsei University (Permit number: 7001988–201802-BR-119-01E). The SNUhES31 human embryonic stem cell (hESC) line was obtained from the Institute of Reproductive Medicine and Population, Medical Research Center, Seoul National University Hospital, South Korea. The hPSCs were cultured on 10 μg/mL mitomycin-C (Roche, Mannheim, Germany)-treated mouse embryonic fibroblasts and maintained in hESC medium composed of 20% knock-out serum replacement (Life Technologies, Carlsbad, CA, USA), 1% minimum essential medium-nonessential amino acids (MEM-NEAA) (Life Technologies, Carlsbad, CA, USA), 1% glutamax (Life Technologies, Carlsbad, CA, USA), and 7 μL/L β-mercaptoethanol (Sigma-Aldrich, St. Louis, MO, USA) in Dulbecco’s modified Eagle medium/nutrient mixture F-12 (DMEM/F-12; Thermo Fisher Scientific, Waltham, MA, USA) with 20 ng/ mL of basic fibroblast growth factor (R&D Systems, Minneapolis, MN, USA). After stabilization of stem cell, stem cell was cultured with feeder-free conditions. For the feeder-free hPSC culture, hPSCs were detached from feeder cells using 1 mg/mL dispase (Life Technologies, Carlsbad, CA, USA) and cultured in Essential 8 medium (Life Technologies, Carlsbad, CA, USA) on Geltrex (Life Technologies, Carlsbad, CA, USA)-coated culture plates. hPSCs were subcultured as small clusters every 4 days using 0.5 mM ethylenediaminetetraacetic acid (EDTA) solution. The cells overexpressing RFX3, 4 and BAMN factor were cultured in Essential 8 medium or neural medium composed of 1% N-2 supplement (Life Technologies, Carlsbad, CA, USA) and 1% MEM-NEAA in DMEM/F-12.

### NPC differentiation and culture conditions

To differentiate hiPSCs into NPCs, embryoid bodies (EBs) were generated by culturing human hiPSCs for 5–6 d on non-adherent Petri dishes in Essential 8 (Invitrogen, Waltham, MA, USA) supplemented with 5 μM dorsomorphin (Sigma-Aldrich, St. Louis, MO, USA) and 5 μM SB431542 (Sigma-Aldrich, St. Louis, MO, USA). The EBs were subsequently attached to a new culture dish coated with Matrigel (BD Biosciences, Franklin Lakes, NJ, USA) containing a neural induction medium composed of DMEM/F-12 medium (Invitrogen, Waltham, MA, USA), 1 × N-2 supplement (Invitrogen, Waltham, MA, USA), and 1 × nonessential amino acids (Invitrogen, Waltham, MA, USA) for 6 days. When neural rosette formation appeared at the center of the EBs, NPCs were collected using a Pasteur pipet [17–19].

### Genetic regulation of the RFX family and BAMN factor using lentiviral vectors

To construct a lentiviral vector overexpressing RFX2, 3, 4, 5, and BMAN factor we subcloned the RFX family gene into the lentiviral vector under the regulation of the cytomegalovirus early enhancer/chicken β-actin (CAG) promoter. Transcript sequences of the RFX family were obtained from the NPCs. Moreover, to generate the heterozygous RFX4 KD cell line using the CRISPR-Cas9 system, the LentiCRISPRv2GFP vector, gifted by David Feldser (Addgene Plasmid # 82416) [20], was used. Lentivirus was constructed after cloning into a CRISPR vector for gRNA targeting RFX4; target sequence 5′-CCACTCCTGCTACTCTGCAA-3′ and 5′-TTATTCCAGCCACACATCTC-3′. Lentiviral capsid vectors were provided by Dr. Yibing Qyang (Yale Cardiovascular Research Center, Yale School of Medicine). For the production of lentiviral particles, RFX family overexpression plasmids were co-transfected with the lentivirus-packaging plasmids (vesicular stomatitis virus G-expressing envelope plasmid) and another plasmid containing the gag, pol, and rev genes (provided by Dr. Yibing Qyang) into HEK293T cells using the X-tremeGene HP DNA transfection reagent (Roche Applied Science, Penzburg, Germany) at 37 °C in a 5% CO_2_ atmosphere for 24 h. The virus-containing medium was collected daily for 3 days after transfection and concentrated via ultracentrifugation at 55,200 × *g* at 4 °C for 2 h (Hitachi, Ltd., Tokyo, Japan). To transfect hESCs with lentiviral particles, hESCs dissociated into single cells were plated in 24-well plates with a concentrated virus-containing medium at a titer of 2 × 10^7^ IU/mL for 24 h at 37 °C, followed by 2 days of culture. The cells were selected using 2 μg/ mL puromycin (Life Technologies). To verify RFX4 knock out, the expression level was confirmed by western blotting after virus infection, and amplicon DNA sequencing for the sgRNA targeted region was performed by selecting one of the reduced expression line.

### ATAC-seq data processing and analysis

ATAC-seq data from the Gene Expression Omnibus (GEO) (GSE158382) were used. Initially, the quality of reads was checked using “FastQC” and executed the adapter removing process using “Cutadapt” (cutadapt -a CTGTCTCTTATACACATCT -q 10 –minimum-length 36). Processed reads were aligned using “Bowtie2” with the option for accurate mapping (bowtie2 –very-sensitive -x < genomeIndexName > -X 2000) on hg19 reference genome. Duplicate reads were discarded using Picard Mark Duplicate software (version 1.141). Peaks were called using MACS2 (macs2 callpeak -verbose 3 -treatment sample.bam -g hs -B -q 0.05 -extsize 200 -nomodel -shift -100-nolambda-keep-dup all -f BAMPE –outdir dir -call-summits). Subsequently, the iPSC and NPC consensus peak sets were generated by iterative peak merging strategy [21] with default option except for score-per-million (spm) cutoff 2. Differentially accessible regions were identified using DiffBind (v3.8.4) [22] with an adjusted *p* value cutoff of 0.05. Subsequent analysis of differential TF activity was performed using diffTF and chromVAR with the consensus peak set [23, 24]. diffTF were used with bootstrap mode, based on the HOCOMOCCOv11 motif database. chromVAR were used with default settings with filtered collection of human motifs from cisBP database (https://github.com/GreenleafLab/chromVARmotifs). DARs were annotated using ChIPseeker (v1.34.1) with annotation databases generated from UCSC hg19 genome and default setting [25].

### ATAC-seq peak recall strategy to investigate the consistently open region during differentiation

To minimize false positives and negatives when defining chromatin openness, a peak recall strategy was applied [26]. To construct the pseudo-input data, all ATAC peaks from each cell type were merged. Based on all merged ATAC-seq reads, those that overlapped with the merged ATAC peaks were filtered out. Using the pseudo-input data, background ATAC peaks were defined using the MACS2 algorithm. Normalized sequence reads were subsequently calculated for each background peak in iPSC and NPC ATAC-seq data. Based on these data, the background signal was defined as 4.372, resulting in a 0.5% false discovery rate (FDR). The revised ATAC peak groups were defined as common, iPSC-specific, and NPC-specific. For these peak groups, genomic region enrichment of annotation tool (GREAT) analysis was performed to observe the genomic distribution of the peaks in each group [27]. For data visualization in the WashU genome browser, the bamCoverage (deepTools 3.5.0) algorithm was used with the following options: –normalizeUsing CPM, –effective GenomeSize 2,864,785,220. For motif analysis, the HOMER algorithm was used with the following options: peaks.txt hg19 MotifOutput/-size given –mask [28]. All known motif results that showed significant motif enrichment (*p* < 0.01) were collected, and hierarchical clustering was executed with Gene Cluster 3.0 by the value of − log(*p* value). The city-block distance method was used to calculate the similarity metric.

### H3K27Ac ChIP-seq, Hi-C data processing, and Activity-by-Contact model application

H3K27ac ChIP-seq and Hi-C data from the Gene Expression Omnibus (GEO) (GSE158382) were used and all data were processed based on hg19 reference genome. The ABC v0.2.2 pipeline was obtained from the GitHub repository. This began by calling ATAC peaks using MACS2 with a *p*-value cutoff of 0.1, followed by defining the candidate enhancer regions using the makeCandidateRegions.py script, which resizes each peak to 250 base pairs, counts the ATAC-seq reads, and selects the top 150,000 peaks. The blacklisted regions were removed, and the overlapping regions were merged. Enhancer activity was quantified using run.neighborhoods.py script, which counted the ATAC-seq and H3K27ac ChIP-seq reads in candidate enhancer regions, gene bodies, and promoter regions. Finally, the ABC score was calculated using predict.py, which combines information from enhancer and promoter activities with contact frequency data from the Hi-C profile. A default threshold of 0.02 was applied, corresponding to approximately 70% recall and 60% precision.

### ChIP-seq data processing and analysis

RFX4 ChIP-seq data from the Gene Expression Omnibus (GEO) (GSE216481) were used. The quality of reads was checked using “FastQC”. The data processing were conducted with nfcore chip seq pipeline v2.0.0 (https://nf-co.re/chipseq/2.0.0/docs/usage) with default settings (hg19). Peak files were generated by nfcore pipeline with macs2, and those peak files were intersected with NPC’s ABC model output enhancer–promoter interaction bedpe files to produce RFX4 bound enhancer–promoter pairs in bedpe format. The target genes of RFX4 bound enhancers were utilized to proceed DAVID ontology analysis (https://david.ncifcrf.gov/tools.jsp).

### RNA-sequencing data processing and analysis

We obtained publicly available iPSC and NPC RNA-seq dataset (GSE156723) to utilize STAR-RSEM gene expression matrix. For data visualization in the WashU genome browser, bamCoverage (deepTools 3.5.0) was used with default options. For RNA-seq analysis of WT/RFX4 KD ESC and WT/RFX4 KD NPC, three replicates per condition were generated. Sequenced reads were aligned to the hg38 reference using hisat2 (v2.2.1). The count matrix was created with the featureCounts function in the subread package (v2.0.0), which was subsequently used to detect differentially expressed genes (DEGs) with DESeq2 (v1.38.3). The cutoff for DEGs was set as an FDR < 0.01 and log_2_(fold-change) > 1. For gene ontology and pathway analyses, genes were assigned to each ATAC peak group by connecting the ATAC peaks to genes located within ± 5 kb of the peak regions. The R package GOseq and clusterProfiler were used with default options to analyze over- or under-represented gene ontology term gene groups related to common, iPSC-specific, and NPC-specific ATAC peaks. Gene set enrichment test (GSEA) was performed with GSEA v4.3.2 for Windows program with gene set permutation mode with human phenotype ontology terms (c5.hpo.v2023.1.Hs.symbol) from molecular signiures database (MSigDB).

## Results

### Identification of intrinsic factors driving neuronal differentiation from stem cells

To better understand neuronal differentiation, several studies have focused on identifying external factors that promote the differentiation of neuronal cells, and the discovery of BAM factors and NEUROD1 has been a considerable milestone in understanding this process. However, few studies have investigated the mechanism of action of endogenous induction of the expression of these factors in cells. In our previous studies, we examined changes in gene expression and various epigenetic states, such as histone modification, chromatin accessibility, and chromatin interactions, during the differentiation of iPSCs into NPCs [29, 30]. We reanalyzed our data to identify the intrinsic factors that determine the differentiation of stem cells into neuronal cells by integrate them in more analytical methods.

**Fig. 1:**
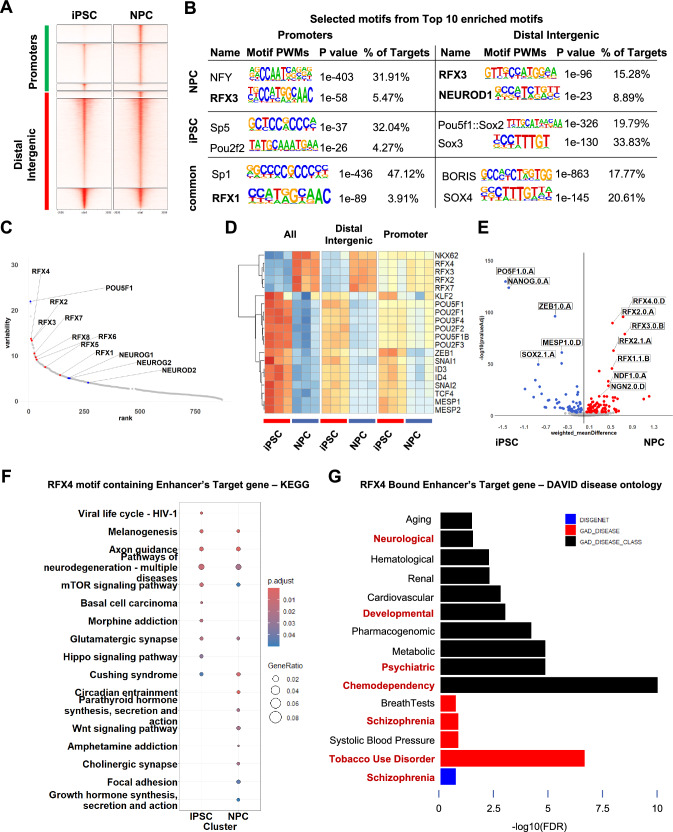
Genome-wide chromatin accessibility analysis during iPSC-to-NPC differentiation. **A** Clustered enriched heatmap between iPSC. and NPC. iPSC and NPC ATAC-seq reads were aligned on the consensus peak set. Clusters from top to bottom; NPC-specific promoter peaks, common promoter peaks, iPSC-specific promoter peaks, NPC-specific distal intergenic peaks, common distal intergenic peaks, iPSC-specific distal intergenic peaks**. B** Top 10 enriched de-novo motifs by HOMER for the iPSC-specific DARs, NPC-specific DARs, and common peaks. **C** Variability ranked-motif plot from chromVAR. The motifs of the RFX family are displayed in red, and conventional proneural factors are displayed in blue. **D** Deviation heatmap from chromVAR. The top 20 most variable motifs were included to generate the heatmap. Among these, NPC-specific enriched motifs consisted of the RFX family. **E** Volcano plot for the differential activity of TF motifs with diffTF analysis. The RFX family shows significant differential activity in NPCs. Conventional proneural factors are also labeled. **F** Dot plot for the KEGG ontology analysis with the RFX4 motif containing enhancer target genes. The common neurobiology-related terms are displayed in blue, and the NPC-specific neurobiology-related terms are displayed in red. G Bar plot for the DAVID disease ontology analysis with the RFX4 bound enhancer target genes. The terms which is related to neuropsychiatric disorders are displayed in red characters

We initially used Diffbind to delineate the differential accessible chromatin state between iPSC and NPC. Consensus peak set was constructed with iterative peak merging strategy[21], whose annotation status was as follow; the promoter region accounted for the largest proportion (38.75%), followed by the intergenic regions (21.37%) (Fig. S1A). Contrary to the annotation results from the consensus peak set, most DARs (|log2FC|> 0 and FDR < 0.05) were found in distal intergenic regions (29.57%), followed by other intronic regions (25.19%) and promoter regions (26.28%) (Fig. S1B). These results suggested that the differentiation stage-specific gene regulatory landscapes are likely driven by enhancer regions. Thereafter, we tried to identify the TFs which might governs each differentiation state (iPSC and NPC). We conducted motif analysis using the HOMER algorithm on stage-specific DARs and common peaks which did not show significant difference between two conditions (Fig. 1A, B). Distinct motif enrichment patterns were observed between the promoter and distal intergenic regions. Among the top 10 enriched *de-novo* motifs, RFX motifs were enriched in common promoter peaks, whereas RFX motifs were enriched in NPC-specific distal intergenic regions and NPC-specific promoter regions. Moreover, NEUROD1, well-known proneural factor, was found to be enriched in NPC-specific distal intergenic regions. These findings, particularly regarding NEUROD1, are consistent with the finding that differentiation-stage-specific gene regulatory networks are largely driven by stage-specific enhancers [14, 31, 32]. Also, RFX family TFs were found to be a possible candidate as an intrinsic factor during NPC differentiation from iPSC.

To examine the differential activity of transcription factors during the differentiation toward neural fate, we employed chromVAR and DiffTF analyses. First, we used chromVAR to identify the TF motifs that exhibited significant variability across different developmental stages. When iteratively merged consensus peakset were used as input, the top-ranked variable motifs included the Oct3/4 (POU5F1) and RFX family (Fig. 1C). The high variability of RFX TF family were largely originated from distal intergenic peaks than promoter peaks (Fig. S1C-D). Furthermore, the deviation score heatmap revealed that among the top 20 variable TFs, the RFX family proteins had a higher score in the NPC group (Fig. 1D), which difference were mainly originated from distal intergenic peaks. To confirm these results, we analyzed ATAC-seq data using diffTF. The diffTF tool combines information on the accessibility or activity of chromatin across the entire genome with potential binding sites for transcription and subsequently calculates the differential activity of TFs between the two conditions. When we used the transcription factor binding site (TFBS) database from HOCOMOCOv11 [33], the most significant NPC-specific TFs were NEUROD1 (NDF1.0.A) and NEUROG2 (NGN2.0.d), which are the well-known proneural factors [31, 34]. As expected, the POU5F1 and NANOG motifs were significantly enriched in iPSCs (Fig. 1E). We observed that the motifs of the RFX family were significantly enriched and were predicted to have higher activity during neuronal differentiation (Fig. 1E). Similar to the chromVAR results, the most of the differential activity of RFX family were originated from distal intergenic peaks (Fig. S1E, S1F). Although the RFX family in promoter peaks showed relatively lower weighted mean difference (Fig. S1F), RFX family showed statistically significant differential activity toward NPC state (adjusted *p* val < 0.001). When the ATAC-seq signal on RFX4 TFBS was visualized, the signals were enhanced in NPCs compared to those in iPSCs, which supports our results (Fig. S1G).

Based on our findings that RFX motifs are considerably accessible in NPCs and that distal intergenic DARs are enriched in RFX family motifs, we hypothesized that RFX-binding motifs in enhancers regulate the NPC state. To test this hypothesis, we integrated ATAC-seq, H3K27ac ChIP-seq, and Hi-C data from iPSCs and NPCs to investigate the connection between enhancers and target genes involved in active chromatin status centered around RFX4. For this analysis, we applied the Activity-by-Contact (ABC) model, which can estimate the activity of enhancers and the physical interactions between enhancers and target genes [24]. We identified the genes connected to the enhancer regions which harbor the RFX motif, and these genes showed significant enrichment of neuronal differentiation-related gene ontology terms, such as axonogenesis and Wnt signaling pathway (Fig. S1H). Specifically, Kyoto Encyclopedia of Genes and Genomes (KEGG) pathway terms including “Axon guidance,” “Pathways of neurodegeneration,” and “Glutaminergic synapse” were significantly enriched in both iPSCs and NPCs, while “Wnt signaling pathway” and “Cholinergic synapse” were significantly enriched only in NPC state (Fig. 1F). These over-representation analyses with Gene ontology biological process (GOBP) and KEGG pathway terms indicated that RFX4 binding enhancers play a specific role in the establishment of the NPC state, while there are also genomic regions that are maintain their accessibility across iPSCs to NPCs. Next, we tried to integrate the RFX4 chip seq dataset generated from induced-NPC (iNPC) [35]. By intersecting between RFX4 chip seq peaks and enhancer-gene pairs in NPC, we could demonstrate that the RFX4 bound enhancer-regulating genes were significantly enriched in various CNS disorders (Fig. 1G). Especially, Psychiatric and Chemo-dependency disorders were to be highly related to the RFX4 bound enhancers, which were composed of schizophrenia, tobacco use disorder. Those results indicate that the RFX4 and RFX4-regulating enhancers might have substantial role in the development of various psychiatric disorders.

### Proneural factor’s promoter peaks are already primed during neural differentiation

**Fig. 2:**
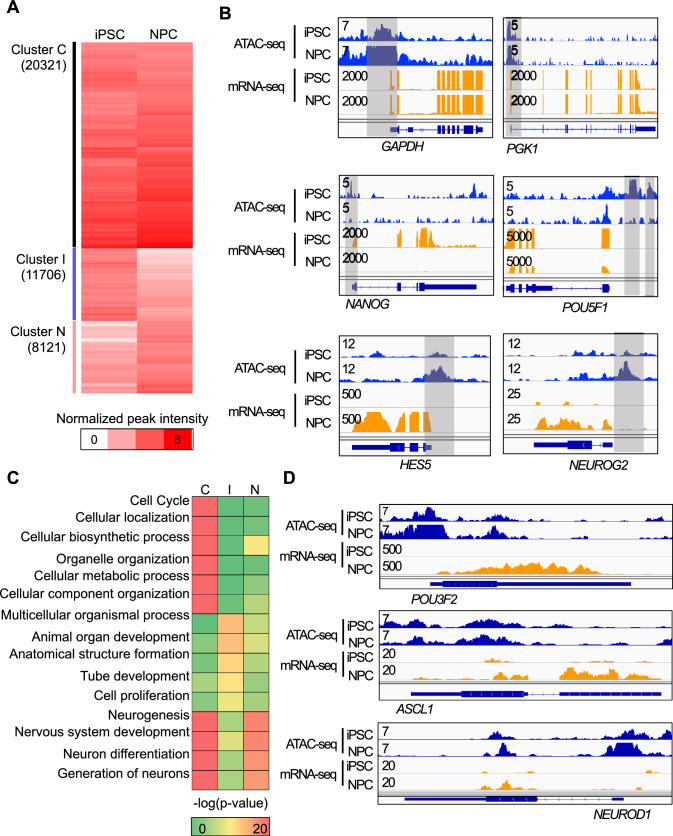
Promoter regions of proneural factors in maintained chromatin accessibility during iPSC-to-NPC differentiation. **A** Heatmap of hierarchical clustering of three ATAC peak clusters (Cluster C: Commonly open, Cluster I: iPSC-specific open, Cluster N: NPC-specific open). The color-coding scale is “Normalized peak intensity”. **B** Graphical presentation of ATAC-seq and RNA-seq signal for three clusters in the WashU genome browser. Representative gene regions of Cluster C (top), Cluster I (middle), and Cluster N (bottom). The intensity of ATAC-seq and mRNA-seq read counts was calculated using bamCoverage and normalized with unique mapped read counts (scale = 107/ < each sample’s total unique reads >). **C** Heatmap of gene ontology analysis results of three clusters. *C* indicates Cluster C, *I* indicates Cluster I, and N indicates Cluster N. Gene ontology analysis was executed using GOseq, R package with the default setting. The color-coding scale is − log10(*p*-value). The *p* value was calculated for over-represented GO terms. **D** Graphical representation of ATAC-seq and RNA-seq signals for proneural factors, POU3F2 (top), ASCL1 (middle), and NEUROD1 (bottom), included in Cluster C. The intensity of ATAC-seq and mRNA-seq read counts was calculated using bamCoverage and normalized using unique mapped read counts (scale = 107 / < each sample’s total unique reads >)

We examined the significant differences in chromatin accessibility between iPSCs and NPCs. Differential analysis using a consensus peak set and DARs revealed differences between the enhancer-driven regulatory networks of iPSC and NPC. However, it is hard to distinguish between closed or opened chromatin states with ‘differential’ accessibility analysis. Also, RFX motif were significantly enriched in non-differential, common promoter peaks. In this regard, in addition to analyzing differentially accessible chromatin regions, we aimed to intensively analyze regions that remain consistently open during the differentiation process. We dichotomized the chromatin state into “open” or “closed”. We used a peak recall process that compared all ATAC peaks to pseudo-inputs to set a standard for the background signal of peak intensities (Fig. S2A) [26]. We identified 20,321 common open regions (Cluster C), 11,706 iPSC-specific accessible regions (Cluster I), and 8,121 NPC-specific accessible regions (Cluster N) (Fig. 2A). Using Genomic Regions Enrichment of Annotations Tool (GREAT) analysis (version 4.0.4), we found that 45% of Cluster C was located within ± 5 kb from transcription start sites (TSS). In contrast, only 7% of Cluster I and 10% of Cluster N were observed within ± 5 kb from TSS (Fig. S2B–D). This indicated that the accessible regions in Cluster C were more closely related to promoter regions than those in the other clusters. In Cluster C, we observed a few housekeeping genes, such as GAPDH and PGK1. Genes regulating stem cell maintenance, such as POU5F1 and NANOG, were in Cluster I, and genes such as HES5 and NEUROG2, which play important roles in neuronal differentiation, were observed in Cluster N. To determine the relationship between chromatin accessibility and the expression of associated genes, we generated RNA-seq data for iPSC and NPC states. A positive correlation was observed between the expression of the genes of interest and ATAC peak intensity (Fig. 2B). We also conducted a gene ontology analysis for each cluster using GOseq using the default settings [36]. To analyze the possible direct impact of ATAC peaks on genes, we selected genes within ± 5 kb of the ATAC peaks with a > twofold intensity change, except for Cluster C (Fig. 2C). General organ development- and cell proliferation-related terms were present in Cluster I. As expected, the genes associated with Cluster N were highly related to neuronal developmental processes. Genes in Cluster C were involved in general cellular functions, such as the cell cycle, cellular metabolic processes, and organelle organization. Notably, genes assigned to Cluster C also showed a strong relationship with neuronal differentiation process terms, similar to that of Cluster N. We further investigated the genes in Cluster C and found that proneural factors such as POU3F2, ASCL1, and NEUROD1 were included. Furthermore, the promoter regions of these genes were already accessible in iPSCs and exhibited increasing expression patterns, while maintaining accessibility during NPC differentiation (Fig. 2D). These results suggest the following hypotheses: First, the processed ATAC-seq data accurately represents the characteristics of each cluster. Second, Cluster N- and Cluster C-related genes are involved in the neuronal differentiation process without a change in chromatin accessibility.

### RFX motifs are enriched in primed peaks during NPC differentiation

**Fig. 3:**
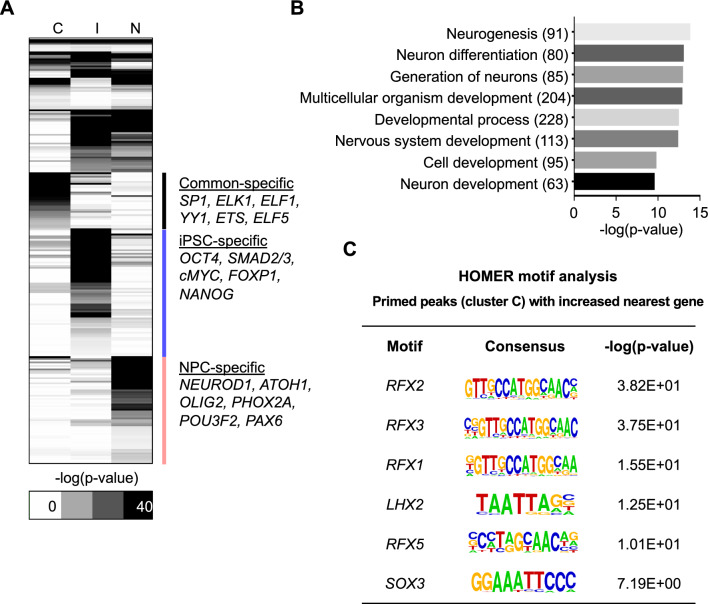
Motifs of the RFX family are enriched in promoters of induction genes in commonly open chromatin regions. **A** Heatmap of hierarchical clustering of highly enriched TFs from three ATAC peak clusters (*C* Cluster C, *I* Cluster I, *N* Cluster N). The color-coding scale is “ − log10(*p* value).” TFs that had a significant *p* value (*p* < 0.01) in at least one cluster were selected. B Gene ontology analysis results of induction genes in Cluster C. Gene ontology analysis was executed using GOseq, R package with the default setting. **C** Enriched motifs for promoter regions of induction genes in Cluster C. Motif analysis was executed using HOMER with a specific setting

We used HOMER to perform a TFBS motif enrichment analysis to identify which TFs were specifically enriched in each cluster. First, we selected TF motifs that showed significant enrichment (*p* < 0.01) in each cluster and then used a hierarchical clustering analysis method to identify specific enrichment patterns. This analysis revealed strong enrichment of TFs crucial for stem cell maintenance, such as POU5F1, NANOG, and c-MYC, in Cluster I. In addition, the TF motifs of NEUROD1, ATOH1, OLIG2, POU3F2, and PAX6, which are important factors in neuronal development, were specifically enriched in Cluster N. For Cluster C, the motifs of general TFs, such as SP1, ELK1, ELF1, and ELF5, were highly enriched (Fig. 3A). Proneural factors were also included in Cluster C in our analysis, which is expected given their roles in neuronal development, including the direct reprogramming of dermal fibroblasts to functional neurons. Thereafter, we performed gene ontology analysis on genes that showed increased expression during iPSC-to-NPC differentiation in Cluster C, confirming significantly enriched neuronal development-related GO terms such as neurogenesis and nervous system development (Fig. 3B). We subsequently investigated which TF motifs were enriched in primed (already opened in iPSC state) regions whose nearest genes are increased in NPC than iPSC state. As a result, strong motif enrichment of RFX family were found, which suggest the promising important roles of RFX family during neuronal development (Fig. 3C).

### RFX4 regulates proneural factors during neuronal differentiation

**Fig. 4:**
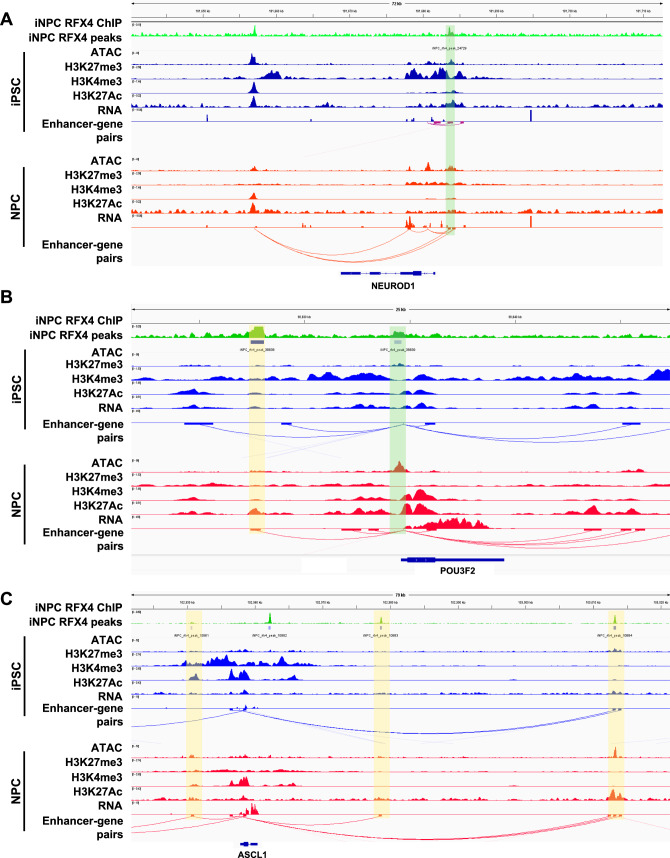
RFX4 directly regulate the NEUROD1 and POU3F2 through promoter and/or enhancer. **A** Genome browser view (IGV) of NEUROD1 locus. Green shade indicates the RFX4 bound promoter regions of NEUROD1 locus. **B** Genome browser view (IGV) of POU3F2 locus. Green shade indicates the RFX4 bound promoter regions of NEUROD1 locus. Yellow shade indicates the RFX4 bound enhancer regions. **C** Genome browser view (IGV) of ASCL1 locus. Yellow shade indicates the RFX4 bound enhancer regions

The integrative analysis of epigenomic data and transcriptomic data above suggest that the RFX family may regulate the NPC-specific enhancers and primed promoter peaks to regulate the neural differentiation process. Indeed, iNPC RFX4 ChIP peak were found in the promoter regions of *NEUROD1* and *POU3F2* locus (Fig. 4A, B). RFX4 bound promoter region in *NEUROD1* locus showed decreased H3K27me3 signal and increased RNA expression in NPC. Also, in *POU3F2* locus, RFX4 bound enhancer who have increased H3K27Ac histone signal in NPC were found to be regulate *POU3F2* by ABC model. In contrast to NEUROD1 and POU3F2, three candidate enhancer regions were found to regulate ASCL1 (Fig. 4C). However, there were no evidence of RFX4 binding event in promoter region.

RFX family proteins consist of eight isoforms (RFX1–RFX8) that regulate genes involved in the cell cycle, DNA repair, and ciliary formation. RFX1, RFX2, RFX3, RFX5, RFX7, and RFX8 are expressed only in the brain, but RFX4 is expressed in both the brain and spinal cord. Also, there have been little evidence to suggest that any RFX protein is involved in the neuronal differentiation from stem cells [37]. When expression patterns of all RFX family genes were analyzed, RFX2, RFX3, and RFX4 showed low expression levels in the iPSC states, with expression induction from a low level in the iPSC state (Fig. S3). Based on these results with the epigenomic analysis, we hypothesized that RFX2, RFX3, and RFX4 are involved in NPC differentiation processes, particularly in regulating the expression of proneural factors.

**Fig. 5:**
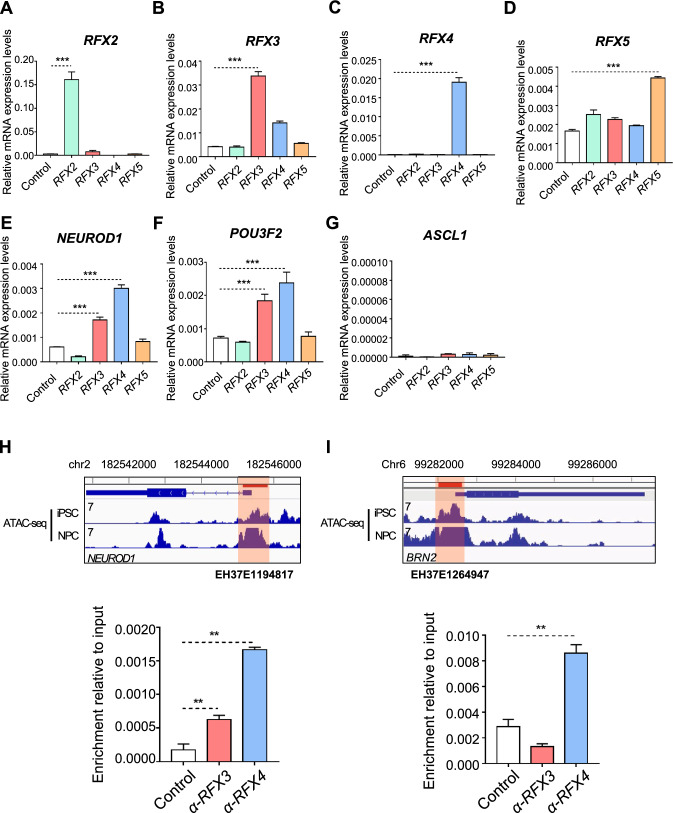
RFX4 activates POU3F2 and NEUROD1 via direct binding of promoter regions. **A**–**D** Bar plots of relative mRNA expression of the candidate RFX family in RFX2-, RFX3-, RFX4-, and RFX5-overexpressing cells 3 days after infection. BAMN refers to BAM-factors-with-NEUROD1. Data are represented as the mean ± S.D. The *p* value is derived using the unpaired *t* test ****p < 0.0001 and ***p < 0.001. **E**–**G** Bar plots of relative mRNA expression of proneural factors 3 days after infection in RFX2-, RFX3-, RFX4-, and RFX5-overexpressing iPSCs. BAMN refers to BAM-factors-with-NEUROD1. Data are represented as the mean ± S.D. The *p* value is derived using the unpaired *t* test ****p < 0.0001 and ***p < 0.001. **H** Target promoter region of chromatin immunoprecipitation (ChIP) followed by RT-qPCR for NEUROD1 (up). Bar plots of relative enrichment to input sample in NEUROD1 targeted qPCR experiments 3 days after infection (bottom). Data are represented as the mean ± S.D. The *p*-value is derived using the unpaired *t* test. ****p < 0.0001 and ***p < 0.001. **I** Target promoter region of chromatin immunoprecipitation (ChIP) followed by RT-qPCR for POU3F2 (up). Bar plots of relative enrichment to input sample in POU3F2 targeted qPCR experiments 3 days after infection (bottom). Data are represented as the mean ± S.D. The *p*-value is derived using the unpaired t test. ****p < 0.0001 and ***p < 0.001

We proceeded to overexpress RFX2, RFX3, RFX4, and RFX5 (as a control) in stem cells using the lentiviral system and investigated the expression of each gene using RT-qPCR. At 3 days post-infection (DPI 3), we confirmed the successful overexpression of RFX2, RFX3, RFX4, and RFX5 in stem cells (Fig. 5A–D). To determine whether these candidate RFX genes could regulate proneural factors, we assessed the expression patterns of NEUROD1, POU3F2, and ASCL1 under RFX overexpression. Notably, overexpression of RFX3 and RFX4 induced both NEUROD1 and POU3F2 but not ASCL1 (Fig. 5E–G) Moreover, overexpression of RFX2 and RFX5 had no effect on the expression of proneural factors. These results suggest RFX3 and RFX4 can potentially regulate proneural factors and drive neuronal differentiation. Next, we investigated whether RFX3 and RFX4 regulated proneural factors by directly binding to the promoter regions of each gene. We performed ChIP experiments for RFX3- and RFX4-overexpressing samples using antibodies against RFX3 and RFX4 and calculated the enrichment ratio over input samples with RT-qPCR primers that targeted promoter regions defined in ENCODE Candidate Regulatory Elements. We confirmed that the candidate regions showed high accessibility in iPSC and NPC states, indicating an optimal chromatin environment for specific TFs to operate. Finally, we found that in contrast to RFX3, RFX4 was significantly bound to the promoter regions of both NEUROD1 and POU3F2 (Fig. 5H, I). Collectively, these data suggest that RFX4 possibly induce NEUROD1 and POU3F2 expression by directly targeting their promoter regions.

### RFX4 promotes neuronal lineage differentiation from stem cells

We investigated the role of RFX4 in neuronal development and its ability to enhance the expression of proneural factors. Following successful lentiviral infection (Fig. S4A), we examined the cellular phenotype after 3 days. however, no discernible changes were observed in the control and RFX3- and RFX4-overexpressing cells (Fig. S4B). Subsequently, we evaluated the expression of crucial stem cell maintenance markers such as OCT4, NANOG, and TRA-1–60 (Fig. S4C, D). We found that only RFX4-overexpressing cells exhibited reduced expression of stem cell markers, indicating that RFX4 impedes stem cell maintenance and induces changes in differentiation status. Next, we investigated which primary germ layer is affected by RFX4 overexpression with three germ layer-specific markers (MIXL1 and Brachyury T as mesoderm markers, SOX1 and PAX6 as ectoderm markers, and GATA6 and SOX17 as endoderm markers) (Fig. S4E–J). Ectoderm/NPC markers (SOX1 and PAX6) were higher in RFX4-overexpressing cells than in RFX3-overexpressing cells, whereas endoderm and mesoderm markers exhibited little or no change. In addition, RFX4-overexpressing cells failed to maintain stem cell morphology through long-term culture and showed a pattern of differentiation (data was not shown). These results indicate that RFX4 overexpression might induce differentiation of stem cells toward neural fate.

**Fig. 6:**
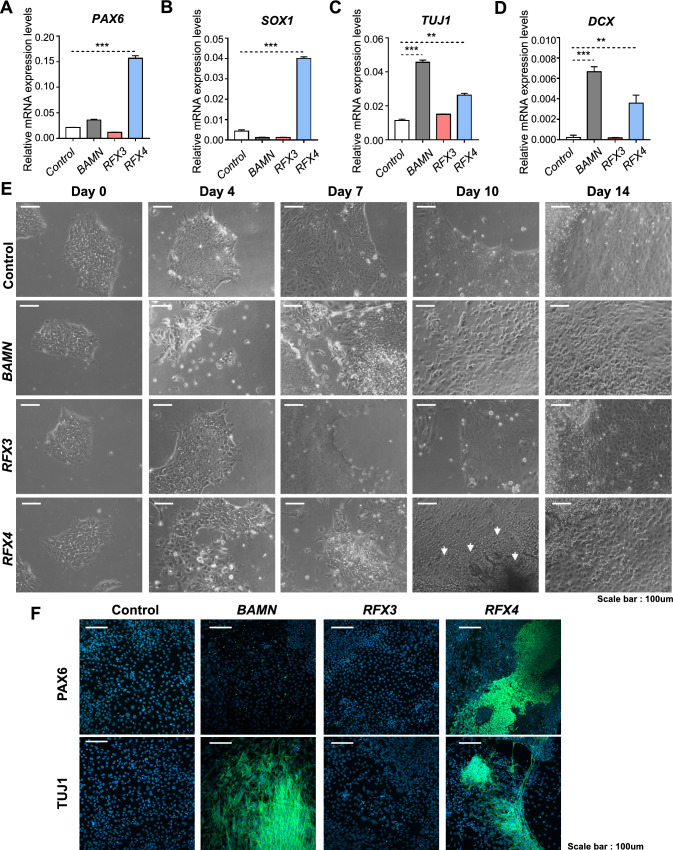
RFX4 can drive iPSC to neuronal lineage. **A**–**D** Bar plots of the relative mRNA expression level of neuronal progenitor cell markers (PAX6 and SOX1) and neuron markers (TUJ1 and DCX) 14 days after overexpression and neural medium culture. Data are represented as the mean ± S.D. The *p*-value is derived using the unpaired *t* test. ****p < 0.0001. **p = 0.0022. **E** Time course bright field microscope analysis after overexpressing control, BAMN (POU3F2, ASCL1, MYT1L with NEUROD1), RFX3 and RFX4. White arrow indicates neuronal rosette-like formations, a unique structure of NPCs. Scale bar indicates 100 µm. **F** Immunocytochemistry data detecting neuronal marker TUJ1 (upper panel) and NPC marker PAX6 (lower panel) after 14 days of culture with neuronal survival media after overexpressing the control vector, BAM factor with NEUROD1, RFX3, and RFX4. Scale bar indicates 100 µm

Next, to compare the neuronal differentiation-driving abilities of RFX4 with known proneural factors, we constructed a BAM-factors-with-NEUROD1 (BAMN)-overexpression system, which drives the direct conversion of fibroblasts to functional neurons. In previous studies, BAMN overexpression in hESCs led to the generation of functional neurons 15 d after induction [13]. Before further analysis, we confirmed the overexpression patterns of each BAMN target gene (Fig S5A–D). After lentiviral induction and 14 days of culture in a neural medium, we assessed the RNA expression levels of ectoderm/NPC markers (PAX6 and SOX1) and mature neuronal markers (TUJ1 and DCX) (Fig. 6A–D). Notably, RFX4-overexpressing cells induced the expression of both neuronal progenitor cells and neuronal markers. When comparing BAMN-overexpressing and RFX4-overexpressing cells, we observed that the expression levels of PAX6 and SOX1 were higher in RFX4-overexpressing cells, whereas TUJ1 and DCX exhibited higher expression levels in BAMN-overexpressing cells. In contrast, BAMN-overexpressing cells did not show significantly increased expression of ectoderm/NPC markers. To further compare the neuronal differentiation process between BAMN- and RFX4-overexpressing cells, we assessed the cellular phenotype at different time points (days 0, 4, 7, 10, and 14) after culture in a neural medium. Although no phenotypic changes were observed in the control and RFX3-overexpressing cells, characteristic phenotypes of neurons such as long axons and dendrites were easily detected in BAMN-overexpressing cells on day 4, and these phenotypes became more solidified over the 14-day culture period consistent with previous results (Fig. 6E). Notably, in RFX4-overexpressing cells, neuronal rosette-like structure was detected on day 10 (white arrows in Fig. 6E), and characteristic phenotypes of neurons were detected on day 14. Also, Immunocytochemistry with NPC marker PAX6 and neuronal marker TUJ1 revealed that the BAMN-overexpressing cells are directly differentiated to neuron without NPC stage, but RFX4-overexpressing cells are differentiated to neuron through the NPC stage (Fig. 6F). These findings indicate that RFX4 might be a factor that promotes neural induction into early neural stage.

### RFX4 is intrinsically required for neuronal differentiation

To further confirm the role of RFX4 in neuronal differentiation, we established a heterozygous KD cell line using the CRISPR-Cas9 system in hESCs. RFX4 gene showed highly intolerant to loss-of-function variations in human population databases (gnomAD, pLI scores = 1.00). In addition, according to the report that the mutation of RFX4 shows the characteristics of haploinsufficiency, a heterozygous KD model was created [38]. Prior to constructing the KD cell line, we examined the time-dependent gene expression patterns of RFX4, POU3F2, and NEUROD1 during NPC differentiation. Our results showed that RFX4, POU3F2, and NEUROD1 were expressed on day 5 of NPC differentiation (Fig. S6A–C). Subsequently, western blot indicated successful KD of RFX4 expression in hESCs (Fig. S6D). In addition, we confirmed the genotype of the RFX4 KD cell line as a heterozygous (Fig. S6E). Although we observed no significant differences in the morphologic phenotype between the WT and KD cell lines in the stem cell state, the KD cell lines failed to exhibit neuronal rosette formation, the typical morphology of NPC (Fig. S6F). Using this KD cell line, we generated RNA-seq data to analyze the genome-wide effects of RFX4 depletion. With the highly concordant dataset (Fig. S7A), high degree of similarity between WT and KD hESC were found, which supported the morphologic phenotype data (Fig. S7B). To confirm the reliability of our NPC differentiation model, we analyzed the differentially expressed genes (DEGs) from WT hESC to WT NPC differentiation and confirmed that various gene ontology terms related to neuronal development processes were highly enriched (Fig. S7C, D).

**Fig. 7:**
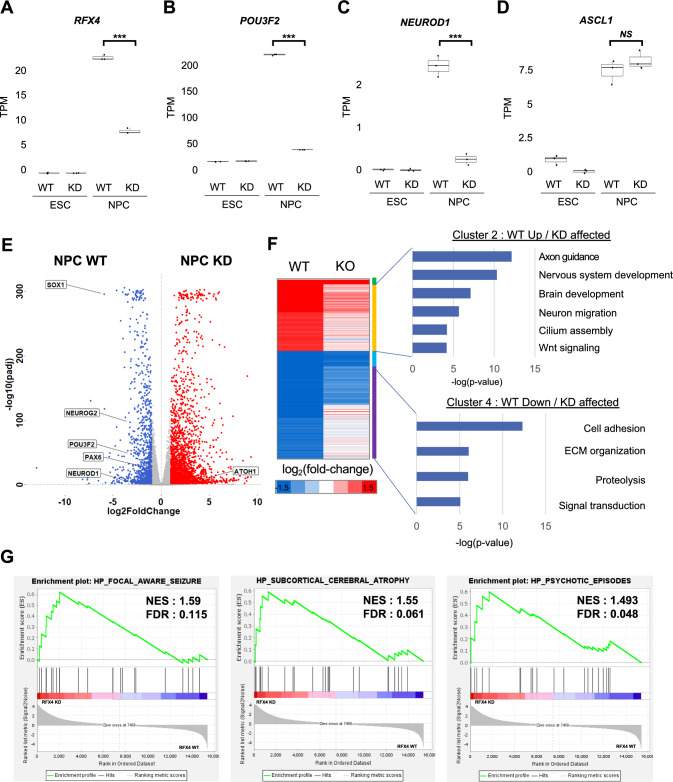
RFX4 is involved in the neuronal differentiation process. **A**–**D** Box plots of mRNA expression level (TPM) of RFX4, candidate targets of RFX4 (POU3F2 and NEUROD1), and ASCL1, which was not expected to be associated with RFX4. We used the t-test to investigate significance of the difference. ***p < 0.001 and NS. indicates *p* > 0.05. **E** Volcano plots for comparing WT NPC and RFX4 KD NPC. Red dots indicate higher expression in WT NPC and blue dots indicate lower expression in KD NPC. Representative genes were noted in each group. **F** Heat map of hierarchical clustering for genes that are normally up- and downregulated during NPC differentiation. Cluster 2 indicates genes that are upregulated in normal neuronal differentiation and are affected by RFX4 KD. Cluster 4 indicates genes that are downregulated in normal neuronal differentiation and are affected by RFX4 KD. The gene ontology analysis results are also displayed. Please refer to Fig. S7E-F for the information of Cluster1 and Cluster 3. **G** Gene set enrichment analysis (GSEA) results with the RFX4 KD NPC vs. WT NPC. *NES* normalized enrichment score, *FDR* false discovery rate

Next, RFX4 were significantly decreased during the differentiation of KD hESCs into NPCs (Fig. 7A). Surprisingly, POU3F2 and NEUROD1 were failed to be induced in RFX4 KD condition (Fig. 7B, C). However, in case of ASCL1, there was no significant difference between WT NPC and RFX4 KD NPC cell lines, indicating that RFX4 depletion did not affect ASCL1 expression (Fig. 7D). Also, we found that the expression levels of neuronal differentiation genes, such as SOX1, NEUROG2, and PAX6, decreased when RFX4 was depleted (Fig. 7E). Furthermore, we attempted to distinguish between groups of genes affected by RFX4 and those unaffected by RFX4. For this purpose, we first defined the genes induced and repressed in the NPC differentiation process by analyzing the difference in the average TPM of each cell type. Based on this gene set, we performed hierarchical clustering. As a result, four clusters were identified: WT up with KD unaffected (Cluster 1), WT up with KD affected (Cluster 2), WT down with KD unaffected (Cluster 3), and WT down with KD affected (Cluster 4) (Figs. 7F, S7E, F). To elucidate the biological significance of each cluster, we performed a gene ontology analysis. In Cluster 1, the genes were significantly related to normal neuronal differentiation (Fig. S7E). Genes in Cluster 2 showed a relationship with more specific neuronal development and mature neuron-related terms such as axon guidance, brain development, and neuron migration (Fig. 7F). Notably, we also found the term “cilium assembly” in Cluster 2, including a list of related genes such as ACTR2, CEP19, and DYNC2H1. Other RFX family members are involved in the regulation of ciliogenesis. Based on these data, we hypothesized that RFX4 plays a role in regulating neuronal differentiation and ciliary formation. When we examined the genes in Clusters 3 and 4, we observed terms such as signal transduction and cell adhesion, which are related to stem cell maintenance (Figs. 7F, S7F). Not only physiological terms of neuronal differentiation/function but also terms of CNS disorders were related with loss of RFX4 function. GSEA analysis also showed significant enrichment of disease term such as “Focal Aware Seizure”, “Subcortical Cerebral Atrophy”, and “Psychotic Episodes” (Fig. 7G), which are known to be closely associated with the development of the CNS from a pathophysiological perspective [39, 40]. These findings suggest that the defect in neuronal development due to the loss of RFX4 function ultimately has associations with various CNS disorders, especially brain diseases.

## Discussion

Numerous studies have highlighted the significance of pioneering factors in lineage determination, owing to their ability to bind and open heterochromatin regions. Since identifying FOXA and GATA4 as TFs capable of binding to closed chromatin in 2002, researchers have actively researched these pioneering factors and their functional mechanisms. To date, several studies have demonstrated the direct reprogramming potential of pioneering factors, including FOXA and GATA4 for hepatocyte-like lineages, Yamanaka factors such as OCT4, SOX2, and KLF4 for stem cell reprogramming, ASCL1 for neuronal lineages, and PU.1 for macrophage-like lineages.

In melanotrope cell specification, both pioneering and non-pioneering factors are required to establish optimal chromatin accessibility [41]. Proneural factors are related to neuronal differentiation in the determination of neuronal lineage. Proneural genes such as Ascl1, Atoh1, Neurog1, Neurog2, and Neurod1, have a basic helix-loop-helix structure. Among proneural genes, ASCL1 and NEUROG2 can directly bind to inaccessible chromatin regions and facilitate changes in the chromatin accessibility of neuronal differentiation-related genic regions during direct reprogramming [12, 14, 42]. In addition to proneural genes, several researchers have identified a combination of factors that can efficiently induce various functional neurons. For example, BAM factors, which include POU3F2, ASCL1, MYT1L, and NEUROD1, can convert mouse and human dermal fibroblasts into functional neurons with high efficiency. Although POU3F2, NEUROD1, and MYT1L do not possess the characteristics of pioneering factors, they are essential for inducing functional neurons [12]. Furthermore, the co-expression of ASCL1 with mesencephalic factors or midbrain-related TFs can induce specific types of neurons, such as peripheral and functional dopaminergic neurons in the midbrain [43].

To elucidate the mechanism of neuronal lineage determination in pluripotent stem cells, it is important to identify master regulators of proneural genes and BAM factors. In this study, we propose that RFX4 induces phenotypically and molecularly proven neurons by activating POU3F2 and NEUROD1. RFX proteins are defined by a winged-helix-type DNA-binding domain conserved in Caenorhabditis elegans, Drosophila, fish, and humans [37, 44–46]. RFX proteins regulate genes involved in developmental processes and ciliary formation in polarized cells [47, 48]. In humans, eight RFX genes (RFX1-8) play similar or distinct roles in various tissues [37]. Among these genes, RFX4 regulates NPCs and neural tube formation in mice and zebrafish [49, 50]. In addition, RFX4 generates neural stem cells from adult mouse cells [51]. Although the role of RFX4 has been reported in animal models, there have been little evidence of its function in humans, except that human RFX4 is specifically expressed in the brain, spinal cord, and testes [37]. However, recent reports suggests that RFX4 might be associated with not only normal neuronal development but also various neuropsychiatric disorders which has neurodevelopment origins. For example, individuals with suicidal behavior showed significantly higher methylation level in *RFX4* locus [16]. Also, recent GWAS study [52] and cohort study [38] indicate that the RFX family (including *RFX4*) genes are associated with autism, attention-deficit/hyperactivity disorder, and alcohol dependence. Also, according to our findings, RFX4 bound enhancer target genes were enriched in Neurological disease, Psychiatric disease, and schizophrenia terms. Moreover, the transcriptome of RFX4 KD NPC showed potential association toward focal aware seizure, subcortical cerebral atrophy, and psychotic episodes. Those disorders are reported to be associated with the CNS development [39, 40]. In summary, the function of RFX4 and their possible association with the specific disorders are only now being reported.

In this study, we report RFX4 as promising gene which is associated with neuropsychiatric disorders. We propose the mechanism underlying those association as novel role of RFX4 in the neuronal differentiation of human pluripotent stem cells by activating POU3F2 and NEUROD1. Like the BAM factor with NEUROD1 (BAMN)-mediated neuronal induction, RFX4 can also generate neurons; however, the RFX4-mediated neuronal differentiation process involves sequential differentiation steps that undergo neuroectodermal stages in the way that neuron differentiation through the NPC, in contrast to the direct reprogramming induced by BAMN. Furthermore, our initial identification of RFX4’s role emerged from experiments with induced pluripotent stem cells (iPSCs), which demonstrated consistency with the anticipated theoretical framework based on data from embryonic stem cells (ESCs). This is a crucial aspect as it confirms our observations in two distinct categories of pluripotent stem cells. We propose that the logic underlying these findings transcends the specific contexts of iPSCs or ESCs, suggesting its relevance across the broad range of pluripotent stem cells. Further studies might include the action of RFX4 in various time points during the neuronal differentiation. Also, more detailed research which delineate the concise mechanisms underlying the association between RFX4 gene and neuropsychiatric disorders should be conducted in future studies.

Our study reveals a novel role for RFX4 in neuronal differentiation and provides insights into the regulatory mechanisms underlying proneural genes and BAM factors in human pluripotent stem cells. Our findings can contribute to developing new strategies for generating functional neurons for various applications, including disease modeling, drug screening, and cell replacement therapy for neuropsychiatric disorders. We anticipate that these findings will provide a foundation for in-depth investigations into RFX genes, including RFX4.

### Supplementary Information

Below is the link to the electronic supplementary material.

Supplementary file1 (DOCX 1485 KB)

## Data Availability

RNA-seq data for RFX4 KD hESC and NPC were deposited in GEO under the accession number GSE233117 (https://www.ncbi.nlm.nih.gov/geo/query/acc.cgi?acc=GSE233117). We used ATAC-seq, H3K27ac ChIP-seq and Hi-C data deposited in GSE158382 (https://www.ncbi.nlm.nih.gov/geo/query/acc.cgi?acc=GSE158382). For RNA-seq data of wild type human iPSC and iPSC-derived NPC, we utilized data deposited in GSE156723 (https://www.ncbi.nlm.nih.gov/geo/query/acc.cgi?acc=GSE156723). For RFX4 ChIP-seq data of iNPC, we utilized data deposited in GSE216477 (https://www.ncbi.nlm.nih.gov/geo/query/acc.cgi?acc=GSE216477).

## Abbreviations

TFs: Transcription factors
ATAC-seq: Assay for transposase-accessible chromatin sequencing
iPSC: Induced pluripotent stem cell
NPC: Neural progenitor cell
DARs: Differentially accessible regions
HOMER: Hypergeometric optimization of motif enrichment
TFBS: Transcription factor binding site
ChIP-seq: Chromatin immunoprecipitation sequencing
Hi-C: High-throughput chromosome conformation capture
ABC model: Activity-by-Contact model
KEGG: Kyoto Encyclopedia of Genes and Genomes
GREAT: Genomic Regions Enrichment of Annotations Tool
TSS: Transcription Start Site
RT-qPCR: Quantitative reverse transcription polymerase chain reaction
DPI: Day post-infection
ENCODE: The Encyclopedia of DNA Elements
CRISPR-Cas9: Clustered regularly interspaced short palindromic repeats and CRISPR-associated protein 9
hESC: Human embryonic stem cell
DEG: Differentially expressed gene
TPM: Transcripts per million
