# Supplementary material for: RFX4 is an intrinsic factor for neuronal differentiation through induction of proneural genes POU3F2 and NEUROD1
Source: Cell Mol Life Sci. 2024 Feb 22;81(1):99. doi: 10.1007/s00018-024-05129-y (PMC10884155; doi:10.1007/s00018-024-05129-y)
Supplement: Supplementary file 1 — Supplementary file1 (DOCX 1485 KB) [file 18_2024_5129_MOESM1_ESM.docx]

Supplementary Informations for

**RFX4 is an Intrinsic Factor for Neuronal Differentiation through Induction of Proneural Genes POU3F2 and NEUROD1**

| **Number of Informations** | **Legends** |
| --- | --- |
| **Supplementary Materials and Methods** |  |
| **Fig. S1.** | **Genome-wide, integrative analysis of chromatin accessibility, chromatin contacts.** |
| **Fig. S2.** | **Accessible chromatin peak re-calling using background signal.** |
| **Fig. S3.** | **The expression pattern of the RFX family during iPSC to NPC differentiation.** |
| **Fig. S4.** | **The effect of RFX3 and RFX4 overexpression on the markers of the three germ line layers.** |
| **Fig. S5.** | **Overexpression of BAM factors with NEUORD1 : RNA-seq** |
| **Fig. S6.** | **Overexpression of BAM factors with NEUORD1** |
| **Fig. S7.** | **RNA-seq data of RFX4-knockout cell lines** |
| **Table S1.** | **Information of primers used in ChIP RT-qPCR** |
| **Table S2.** | **Information of primers used in RT-qPCR** |

Supplementary Materials and Methods

*Immunocytochemistry*

Cells were fixed in 4% paraformaldehyde (PFA; Junsei Chemical Co., Ltd., Tokyo, Japan) for 2 h at 4°C. For immunofluorescence (IF) staining, the fixed cells were permeabilized with 0.1% (v/v) Triton X-100 (Sigma-Aldrich) in PBST containing 10% normal goat serum (Vector Laboratories, Burlingame, CA, USA) for 1 h at 25°C. The fixed cells were subsequently incubated with the following primary antibodies in PBST containing 2% normal goat serum at 4°C overnight: anti-neuron-specific class III beta-tubulin 1 (anti-TUJ1; rabbit, 1:100, Cell Signaling Technology, Danvers, MA, USA, 5666), anti-Oct4 (mouse, 1:200, Santa Cruz Biotechnology, Dallas, TX, USA, sc-365509), anti-Nanog (rabbit, 1:200, Cell Signaling Technology 3580), and anti-Tra-1-60 (mouse, 1:200, Santa Cruz Biotechnology, Dallas, TX, USA, sc-21705). The fixed cells were washed three times with PBST and incubated with the donkey Alexa Fluor 488-conjugated secondary antibody for 3 h at 25°C. Finally, the fixed cells were washed thrice with PBST, mounted under coverslips on slides using Vectashield mounting medium (Vector Laboratories, Newark, CA, USA), and imaged using a TCS SP5 II confocal microscope (Leica, Wetzlar, Germany).

*Chromatin immunoprecipitation (ChIP) followed by RT-qPCR*

The cells were washed with cold phosphate-buffered saline (PBS) and crosslinked with 1% formaldehyde in PBS for 10 min at 25°C. Crosslinking was stopped by the addition of glycine (final concentration: 125 mM) at 25°C for 5 min and washed twice with cold PBS. Crosslinked samples were resuspended in 100 µL of sodium dodecyl sulfate (SDS) lysis buffer (50 mM Tris-HCl [pH 8], 10 mM EDTA [pH 8], and 1% SDS) with protease inhibitor cocktail (Bio-Rad, Hercules, CA, USA, QTPPI1015). The samples were sonicated (BMS Bioruptor (KRB-01), BMS, Seoul, South Korea) using the following parameters: intensity, high; 30 sec on/30 sec off; and five cycles. After sonication, 900 µL of ChIP dilution buffer (1 M Tris-HCl [pH 8], 10% SDS, 10% Triton X-100, 5.1 M NaCl, 0.5 M EDTA, and protease inhibitor cocktail) was added to the sonicated samples. The RFX3 (Novus Biologicals, Littleton, CO, USA) or RFX4 (Novus Biologicals, Littleton, CO, USA) antibodies was incubated with magnetic beads (Invitrogen, Waltham, MA, USA, Dynabeads M-280 Sheep IgG) for 4 h at 4°C with rotation. The chromatin samples were stored as sonicated samples for use as input samples. Thereafter, the samples were incubated with the RFX3 or RFX4 antibodies and magnetic bead complex for 4 h at 4°C with rotation. After incubation, the bead complex was rinsed with wash buffer (140 mM NaCl, 1 mM EDTA [pH 8], 0.5 mM EGTA [pH 8], 1% Triton X-100, 0.1% SDS, 0.1% sodium deoxycholate, and 10 mM Tris-HCl [pH 8]) four times. After incubation, 3 µL of 10% SDS and 5 µL of Proteinase K (20 mg/mL) were added to the samples. Thereafter, reverse-crosslinking at 68°C was executed for 10 h. The chromatin immunoprecipitated DNA samples were extracted using the MinElute® PCR purification kit (Qiagen, Hilden, Germany). RT-qPCR for the ChIP samples was performed on a LightCycler® 480 II system (Roche, Basel, Switzerland) using the FastStart Essential DNA Green Master Kit (Roche, 06402712001). ChIP-qPCR calculations were based on the percentage input method. The Cq value was adjusted according to the percentage of input, and the relative enrichment was calculated as 2^(Ct(Adjusted input) – Ct(ChIP sample))^. For primer information, see Table S1.

*cDNA synthesis and real-time quantitative polymerase chain reaction (RT-qPCR)*

RNA was extracted using the TRIzol reagent (Life Technologies, Carlsbad, CA, USA). cDNA was synthesized using the iScript cDNA Synthesis kit (Bio-Rad, Hercules, CA, USA, #1708890) following the manufacturer’s protocol. RT-qPCR was performed on a LightCycler® 480 II system (Roche, Basel, Switzerland) using the FastStart Essential DNA Green Master Kit (Roche, Basel, Switzerland, 06402712001). Relative expression was calculated by using the 2^Ct(control gene(GAPDH))-Ct(target gene)^ method and human GAPDH was used as control. For primer information, see Table S2.

*Western blot analysis*

The cells were lysed in a lysis buffer (iNtRON Biotechnology, Seongnam, South Korea) using sonication (Vibra-Cell; Sonics, Newtown, CT, USA) on ice. Cell lysates were separated using 10–12% sodium dodecyl sulfate-polyacrylamide gel electrophoresis and transferred to polyvinylidene fluoride membranes (Millipore, Burlington, MA, USA). The blots were washed with TBST (10 mM Tris–HCl [pH 7.6], 150 mM NaCl, and 0.1% Tween-20; Affymetrix, Santa Clara, CA, USA), blocked with 5% skim milk (Millipore, Burlington, MA, USA) for 1 h, and incubated with primary antibodies at 4°C for 10 h on the shaker. The following primary antibodies were used: rabbit anti-RFX3 (rabbit, 1:1000, Novus Biologicals, Littleton, CO, USA), rabbit anti-RFX4 (rabbit, 1:1000 Novus Biologicals, , Littleton, CO, USA), and mouse anti-β-actin (Santa Cruz Biotechnology, Dallas, TX, USA). The primary antibodies were detected using goat anti-rabbit (1:10,000; Santa Cruz Biotechnology, Dallas, TX, USA) or goat anti-mouse (1:10,000; Santa Cruz Biotechnology, Dallas, TX, USA) IgG-conjugated horseradish peroxidase (HRP). Bands were visualized using an enhanced chemiluminescence solution (Thermo Scientific, Waltham, MA, USA). Images were acquired using an ImageQuant LAS 4000 Mini System (GE Healthcare, Chicago, IL, USA).

Fig. S1. Genome-wide, integrative analysis of chromatin accessibility, chromatin contacts.


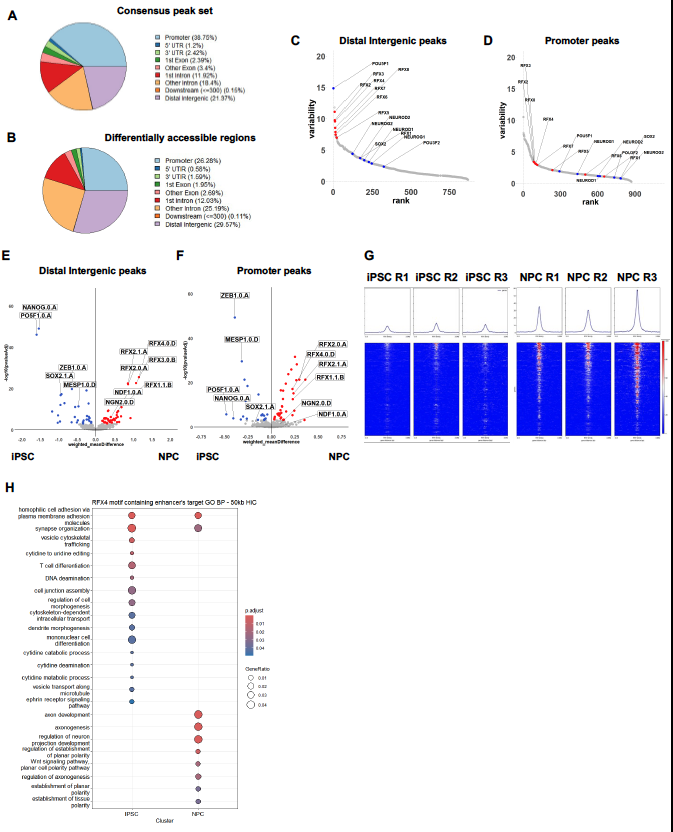


(A-B) Annotation of consensus- and differentially accessible peaks across the genome using ChIPseeker. (C-D) Variability ranked-motif plot from chromVAR with (C) distal intergenic peaks and (D) promoter peaks. The motifs of the RFX family are displayed in red, and conventional proneural factors are displayed in blue. (E-F) Volcano plot for the differential activity of TF motifs with diffTF analysis, with (E) distal intergenic peaks and (F) promoter peaks. (G) The enriched heatmap for RFX motif containing regions. Each row represents the RFX motif-containing peaks. The replicates and reads from ATAC BAM files of each condition were aligned to the reference peaks using DeepTools. The RFX family shows significant differential activity in NPCs. Conventional proneural factors are also labeled. (H) The dotplot for comparative gene ontology analysis with GO:BP database from MSigDB. The input genes were the RFX4 motif containing the enhancer’s targets predicted by the ABC (Activity-by-Contact) model.


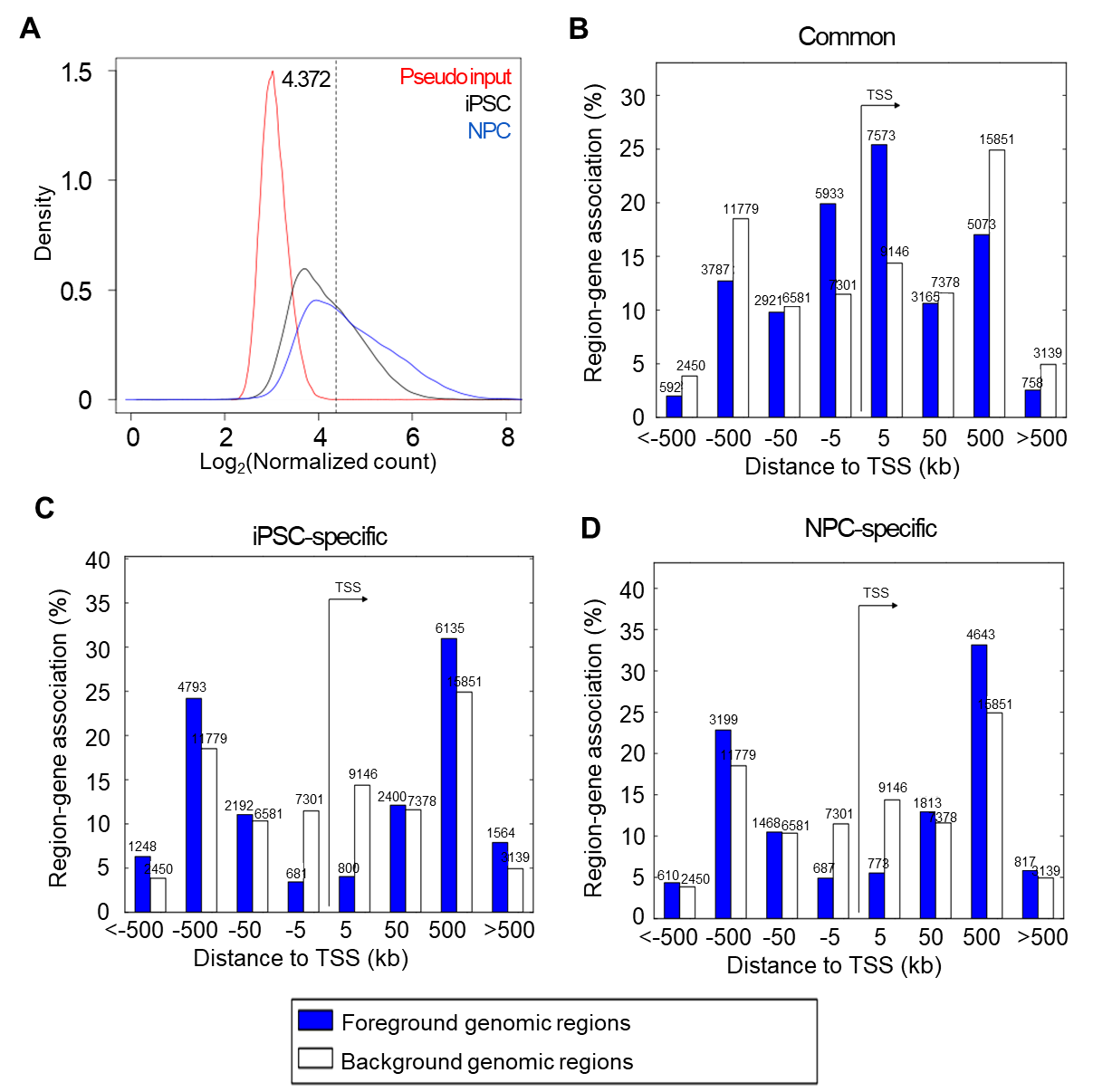
Fig. S2. Accessible chromatin peak re-calling using background signal.

(A) Density plot representing normalized counts of ATAC-seq peaks for pseudo input (red), iPSC (black), and NPC (blue). The dotted line indicates the point of background signal resulting in a 0.5% false discovery rate (FDR) for openness of chromatin structure. Location of peaks according to genomic regions from the transcription start site (TSS). Based on the background signal, we defined (B) commonly open peaks, (C) iPSC-specific open peaks, and (D) NPC-specific open peaks. We noted the number of peaks located in each range of genomic position. We defined background genomic regions as all peaks detected on ATAC-seq.


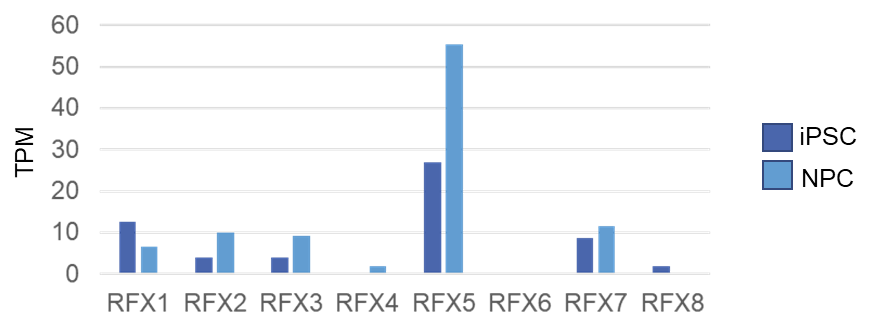
Fig. S3. The expression pattern of the RFX family during iPSC to NPC differentiation.

RNA expression levels (TPM) of RFX family members (RFX1, RFX2, RFX3, RFX4, RFX5, RFX6, RFX7, and RFX8) in iPSC and NPC.


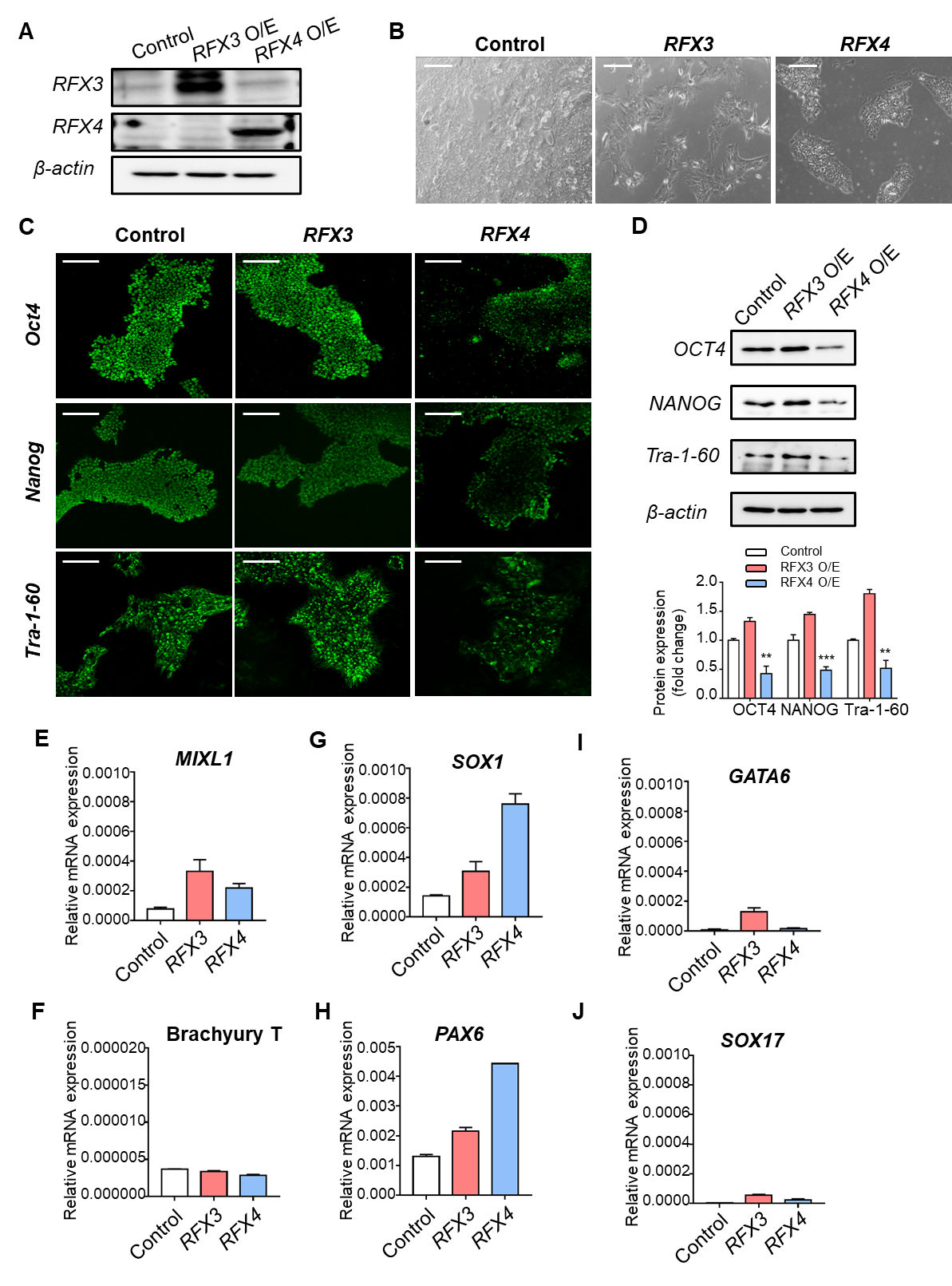
**Fig. S4. The effect of RFX3 and RFX4 overexpression on the markers of the three germ line layers.**

(A) RFX3 and RFX4 protein expression levels in RFX3- and RFX4-overexpressing hPSCs detected via western blot analysis 3 d after infection. (B) Representative images of RFX3- and RFX4-overexpressing hPSCs 3 d after infection. Scale bar = 200 µm. (C) Expression of pluripotent-related proteins such as OCT4, NANOG, and Tra-1-60 were stained via immunocytochemistry in hPSCs 3 d after infection. Scale bar = 100 µm. (D) OCT4, NANOG, and Tra-1-60 expression levels in RFX3- and RFX4-overexpressing hPSCs were detected via western blot analysis 3 d after infection. Mean ± SEM (n = 3). **P <0.01, ***P <0.001 vs. control. (E–J). 3 Mesodermal marker (MIXL1 and Brachyury T), ectodermal marker (SOX1 and PAX6), and endodermal marker (GATA6 and SOX17) expression levels were measured via qPCR in hPSCs with RFX3 and RFX4 overexpression 3 d after infection. Mean ± SEM (n = 3).

**Fig. S5. Overexpression of BAM factors with NEUORD1 : RNA-seq results**


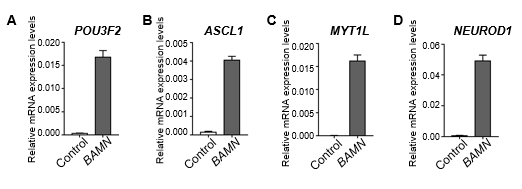


(A–D) Bar plots of the relative mRNA expression of BAMN-overexpressing hPSCs after 3 d of infection. SEM (n = 3).

**Fig. S6. Overexpression of BAM factors with NEUORD1**


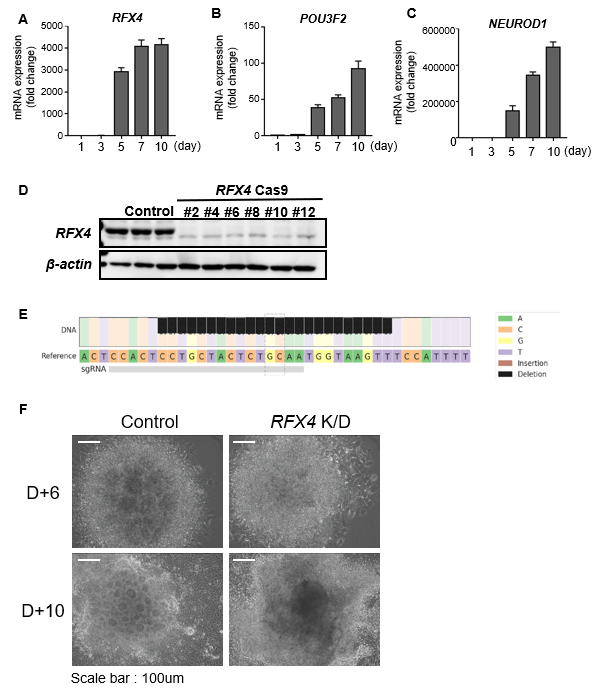


(A–C) Bar plots of relative mRNA expression of RFX4, POU3F2, and NEUROD1 during NPC differentiation derived from hPSCs in a time-dependent manner (days 1, 3, 5, 7, 10). (D) Western blot analysis of RFX4 expression in hPSCs transduced with nontargeting control gRNA with Cas9 and the RFX4-targeting gRNA with Cas9 (RFX4 Cas9) lentivirus. (E) Result of deep DNA sequencing for RFX4 sgRNA targeted regions. At each base in the reference amplicon, the percentage of each base as observed in sequencing reads is shown (A = green; C = orange; G = yellow; T = purple). Black bars show the percentage of reads for which that base was deleted. (F) Representative images of NPC differentiation with Control and RFX4 K/O cell lines on days 6 and 10. Scale bar = 100 µm.

Fig. S7. RNA-seq data of RFX4-knockout cell lines


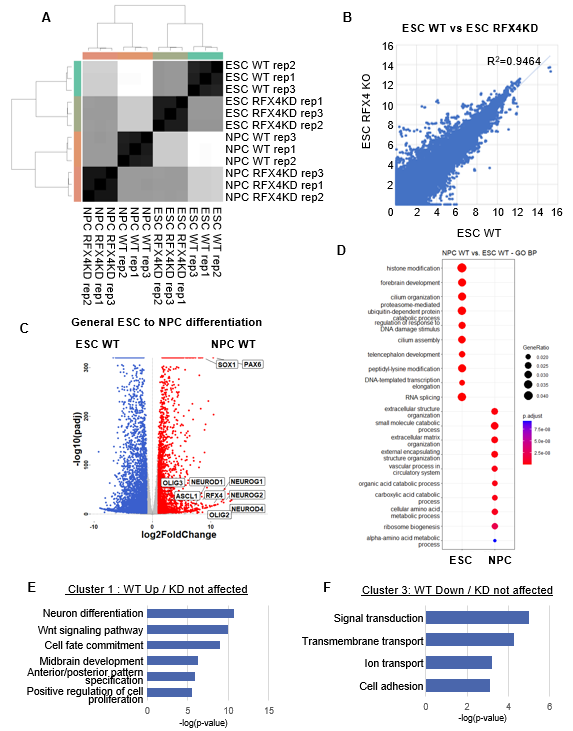


(A) The sample distance heatmap for the generated RNA-seq libraries. (B) The scatterplot representing the significant correlation (R^2^ = 0.9464) between ESC WT libraries and ESC-RFX4 KD libraries. (C) The volcano plot of differentially expressed genes between the ESC WT and NPC WT. The well-known proneural factors were upregulated under NPC WT conditions. (D) The dot plot for the comparative gene ontology analysis with GO Biological Process terms between ESC WT and NPC WT. (E-F) The gene ontology analysis results of (E) Cluster1 and (F) Cluster 3 which is define in Fig 7.

Table S1. Information of primers used in ChIP RT-qPCR

| Gene | Primer sequence |
| --- | --- |
| NEUROD1 | F: CAT GCG CCA TAT GGT CTT CC |
|  | R: AGA TGG GCC ACT TTC TTC TGG |
| POU3F2 | F: TAG CTC TGC GCC AAT CAG T |
|  | R: CGC GCC CTT TGA TTT ACG TG |
| F, Forward; R, Reverse | |

Table S2. Information of primers used in RT-qPCR

| Gene | Primer sequence |
| --- | --- |
| RFX2 | F : CTG CGA CCA CAT CCT CTA CCA G |
|  | R : GCT CTT GGC AAA GTT ACG GAT GG |
| RFX3 | F : AGG AAG TGG TCA ACA GAC AGG C |
|  | R : GGT AGT ACC ATC TGG CAG AGA AG |
| RFX4 | F : CGA GTC CTA CAT TGA GTG GCT G |
|  | R : GTG CCG AAA CAG GAC CAC ATC A |
| RFX5 | F : CAC TGA CAC CTG TCT GCC AAA G |
|  | R : CCT TCG AGC TTT GAT GTC AGG G |
| NEUROD1 | F : GGT GCC TTG CTA TTC TAA GAC GC |
|  | R : GCA AAG CGT CTG AAC GAA GGA G |
| POU3F2 | F : GTG TTC TCG CAG ACC ACC ATC T |
|  | R : GCT GCG ATC TTG TCT ATG CTC G |
| ASCL1 | F : TCC CCC AAC TAC TCC AAC GA |
|  | R : GCG ATC ACC CTG CTT CCA AA |
| MYT1L | F : AGG CAC AGG AAA AGC ACC AGA G |
|  | R : TTG GAA CGC GGC GTA GTT GTG G |
| PAX6 | F : CTG AGG AAT CAG AGA AGA CAG GC |
|  | R : ATG GAG CCA GAT GTG AAG GAG G |
| SOX1 | F : GAG TGG AAG GTC ATG TCC GAG G |
|  | R : CCT TCT TGA GCA GCG TCT TGG T |
| TUJ1 | F : TCA GCG TCT ACT ACA ACG AGG C |
|  | R : GCC TGA AGA GAT GTC CAA AGG C |
| DCX | F : TAT GCG CCG AAG CAA GTC TCC A |
|  | R : CAT CCA AGG ACA GAG GCA GGT A |
| MIXL1 | F : CCC GAC ATC CAC TTG CGC GAG |
|  | R : GGA AGG ATT TCC CAC TCT GAC G |
| GATA6 | F : GCC ACT ACC TGT GCA ACG CCT |
|  | R : CAA TCC AAG CCG CCG TGA TGA A |
| Brachyury T | F : CCT TCA GCA AAG TCA AGC TCA CC |
|  | R : TGA ACT GGG TCT CAG GGA AGC A |
| GAPDH | F : GTC TCC TCT GAC TTC AAC AGC G |
|  | R : ACC ACC CTG TTG CTG TAG CCA A |
| F: Forward, R: Reverse | |
